# A novel histopathologic finding in the Descemet's membrane of a patient with Peters Anomaly: a case-report and literature review

**DOI:** 10.1186/s12886-015-0131-y

**Published:** 2015-10-23

**Authors:** Wei Ni, Wei Wang, Jing Hong, Pei Zhang, Cong Liu

**Affiliations:** Department of Ophthalmology, Peking University Third Hospital, Beijing, 100191 China; Key Laboratory of Vision Loss and Restoration, Ministry of Education, Beijing, China

**Keywords:** Peters anomaly, Descemet's membrane, Anterior segment dysgenesis, Corneal opacity

## Abstract

**Background:**

Peters anomaly is a rare developmental abnormality of the anterior segment of the eye and is one of the main causes of congenital corneal opacities. Typically, histopathology of Peters anomaly shows immature or absent Descemet’s membrane and attenuated endothelial cells in the area of the corneal opacity, in addition to thinning or absence of Bowman’s membrane and defects in the posterior stroma. In this report, we present a novel histopathological finding, which has not been previously reported, in the Descemet's membrane of a patient who is clinically diagnosed with Peters anomaly.

**Case presentation:**

A 7-years old female child with developmentally delayed was born of a normal pregnancy, labor, and delivery. Apparent bilateral corneal opacifications were present at birth. On ophthalmologic examination, the child had a visual acuity of FC/20 cm in the right eye and that of FC/10 cm in the left one. Horizontal nystagmus and congenital cataract were found in both eyes. Slit-lamp examination revealed bilateral central corneal opacities which covered the iris and pupils. High-frequency UBM and AS-OCT both showed a shallow anterior chamber with multiple areas of iridocorneal adhesions and no corneal lenticular touch in each eye. A corneal specialist performed a penetrating keratoplasty with extra-capsular cataract extraction and intraocular lens implantation. Histopathologic procedures were conducted on the host corneal button, including Hematoxylin-Eosin stain and Periodic Acid-Schiff stain. All the sections were examined by light microscopy.

**Conclusion:**

The “multiple-layer” structure of the Descemet’s membrane described in our case has not been reported before as in association with abnormalities of the cornea tissues in Peters anomaly. Such pathological finding need to be reported to enhance further understanding of the special structure of Descemet's membrane as an abnormality during embryogenesis and neural crest cell differentiations.

## Background

Peters anomaly is a rare developmental abnormality of the anterior segment of the eye, either sporadic or inherited. It is one of the main causes of congenital corneal opacities [1]. Typically, histopathology in Peters anomaly shows immature or absent Descemet’s membrane and attenuated endothelial cells in the area of the corneal opacity, in addition to thinning or absence of Bowman’s membrane and defects in the posterior stroma [2]. Corneal opacification is bilateral in approximately 80 % of cases [1]. The opacity is caused by a defect in the underlying corneal endothelium and the Descemet membrane. In this case, we present a novel histopathological finding, which has not been previously reported, in the Descemet's membrane of a patient who is clinically diagnosed with Peters anomaly.

## Case presentation

**Fig. 1:**
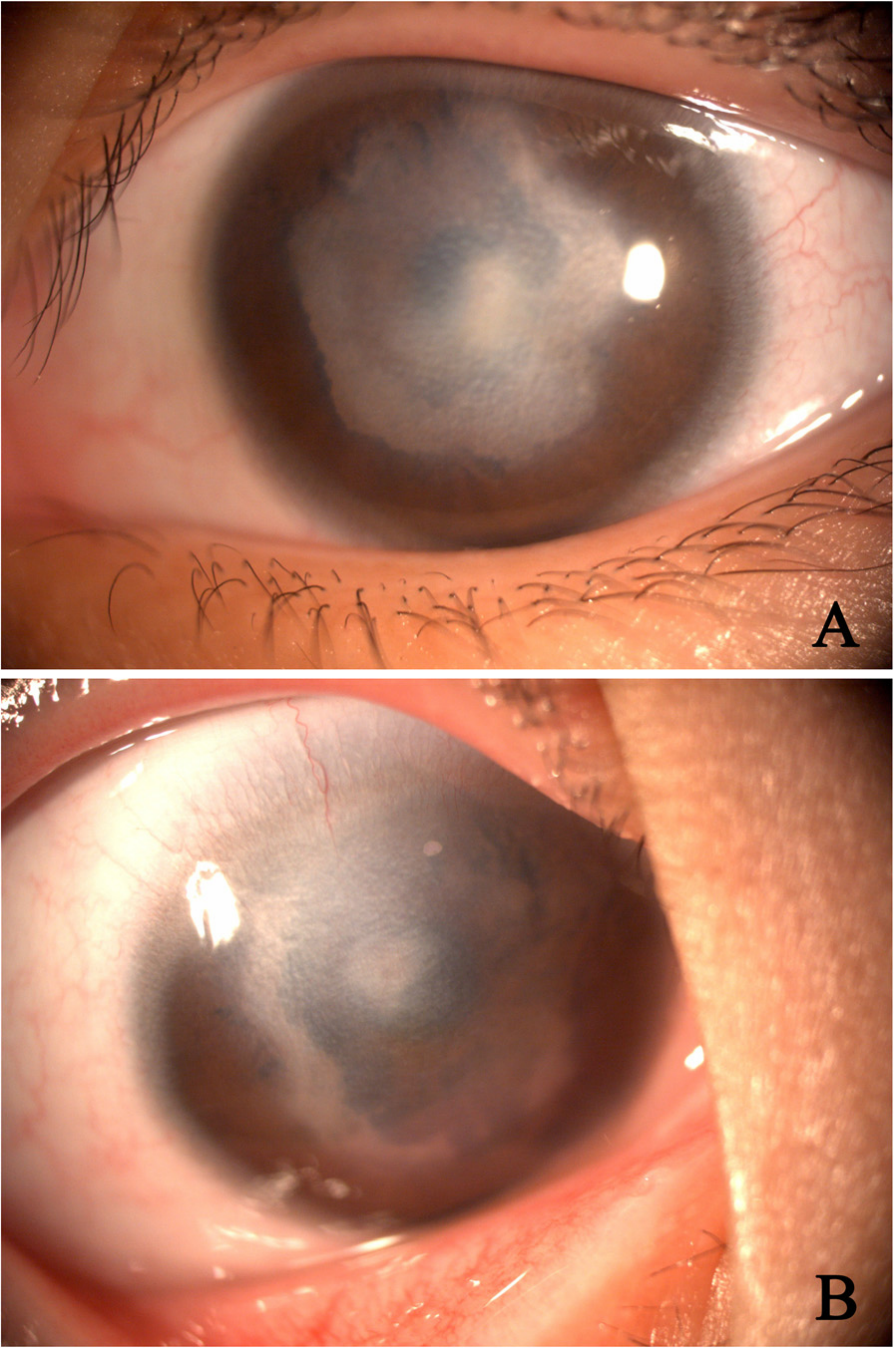
Slit-lamp photographs of the anterior segment of the patient. **a** In the left eye, central corneal opacity, corectopia, anterior synechiae and whitish lens were observed. **b** In the right eye, central corneal opacity, corectopia, anterior synechiae and whitish lens were observed

**Fig. 2:**
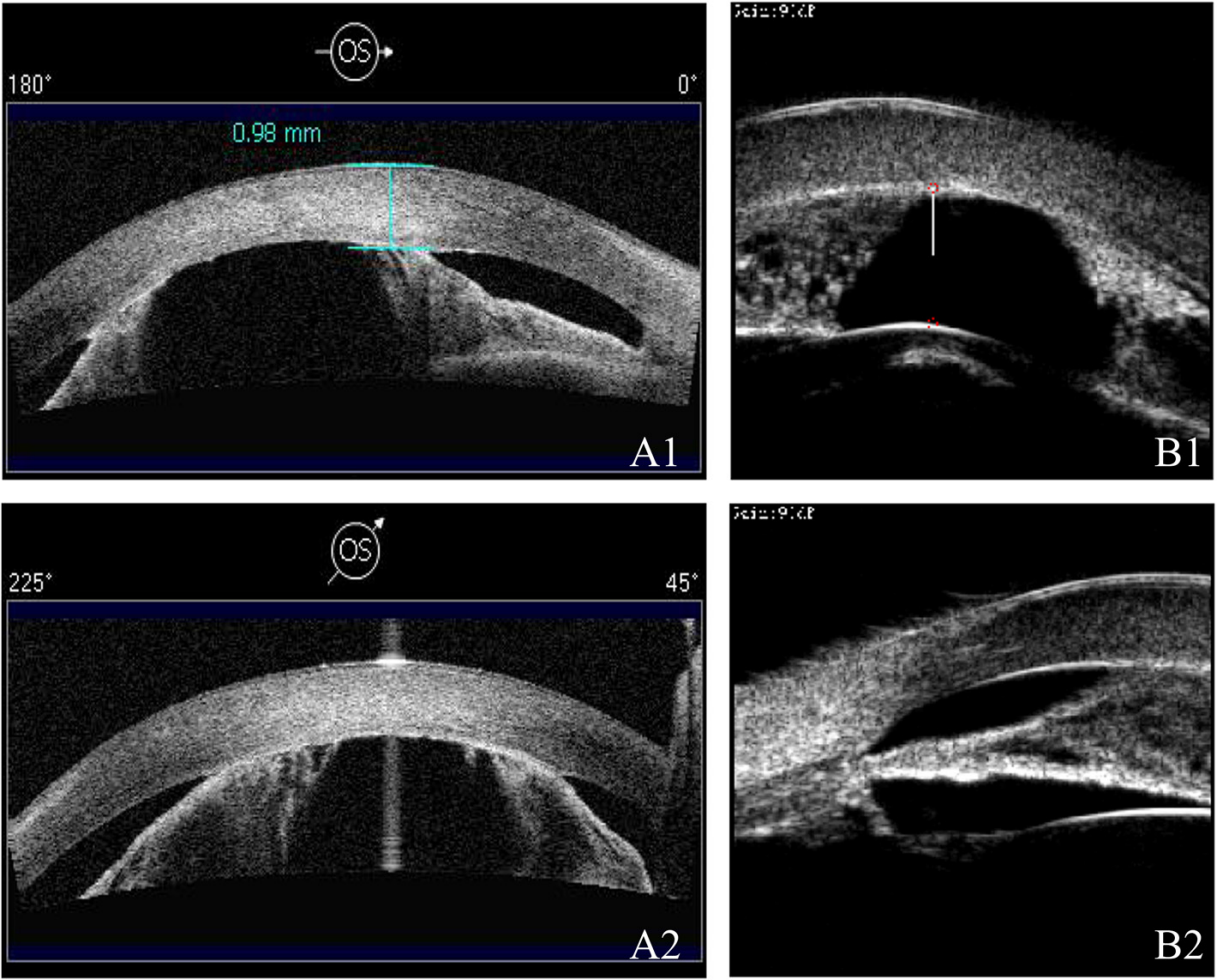
Images by AS-OCT (**a**) and UBM (**b**) of the left eye. *A1*, *2*, AS-OCT showed a shallow anterior chamber with anterior synechiae in the left eye. *B1*, *2*, High-frequency UBM showed a shallow anterior chamber with anterior synechiae and there was no corneal lenticular touch in the left eye

A 7-years old female child with developmentally delayed was born of a normal pregnancy, labor, and delivery. There were no maternal infections and other illnesses detected during the pregnancy as well as the perinatal period, respectively. Ocular anomalies (apparent bilateral corneal opacifications) were present at birth. The infant did not show evidence of tearing, blepharospasm, photophobia, drainage, or ocular redness. On ophthalmologic examination, the child had a visual acuity of FC/20 cm in the right eye and that of FC/10 cm in the left one. The intraocular pressure was normal in both eyes. Slit-lamp examination revealed bilateral central corneal opacities which covered the iris and pupils (Fig. 1a, b). The anterior segments appeared abnormal with corectopia and iris cornea-peripheral anterior synechiae. Horizontal nystagmus and congenital cataract were found in both eyes. The central retina could not be examined clearly because of the severe corneal opacity. High-frequency UBM (Fig. 2a) and AS-OCT (Fig. 2b) both showed a shallow anterior chamber with multiple areas of iridocorneal adhesions and no corneal lenticular touch in each eye. The B-type ultrasound scan performed on the patient revealed no masses, vitreous debris, or retinal detachment in either eye.

Clinically, a pediatric ophthalmologist confirmed the Peters Anomaly diagnosis of the patient, who was then referred to a pediatrician for an examination of associated systemic anomalies. However, none was detected. Under this situation, a penetrating keratoplasty with extra-capsular cataract extraction and IOL implantation was performed by a corneal specialist. The cornea tissue underwent histopathological examination. The patient’s visual acuity improved to 20/100 in the left eye and intraocular pressure was 8 mmHg after the surgical procedures. In addition, the parents of this child were also examined and corneal opacity in the left eye was found in her father.

**Fig. 3:**
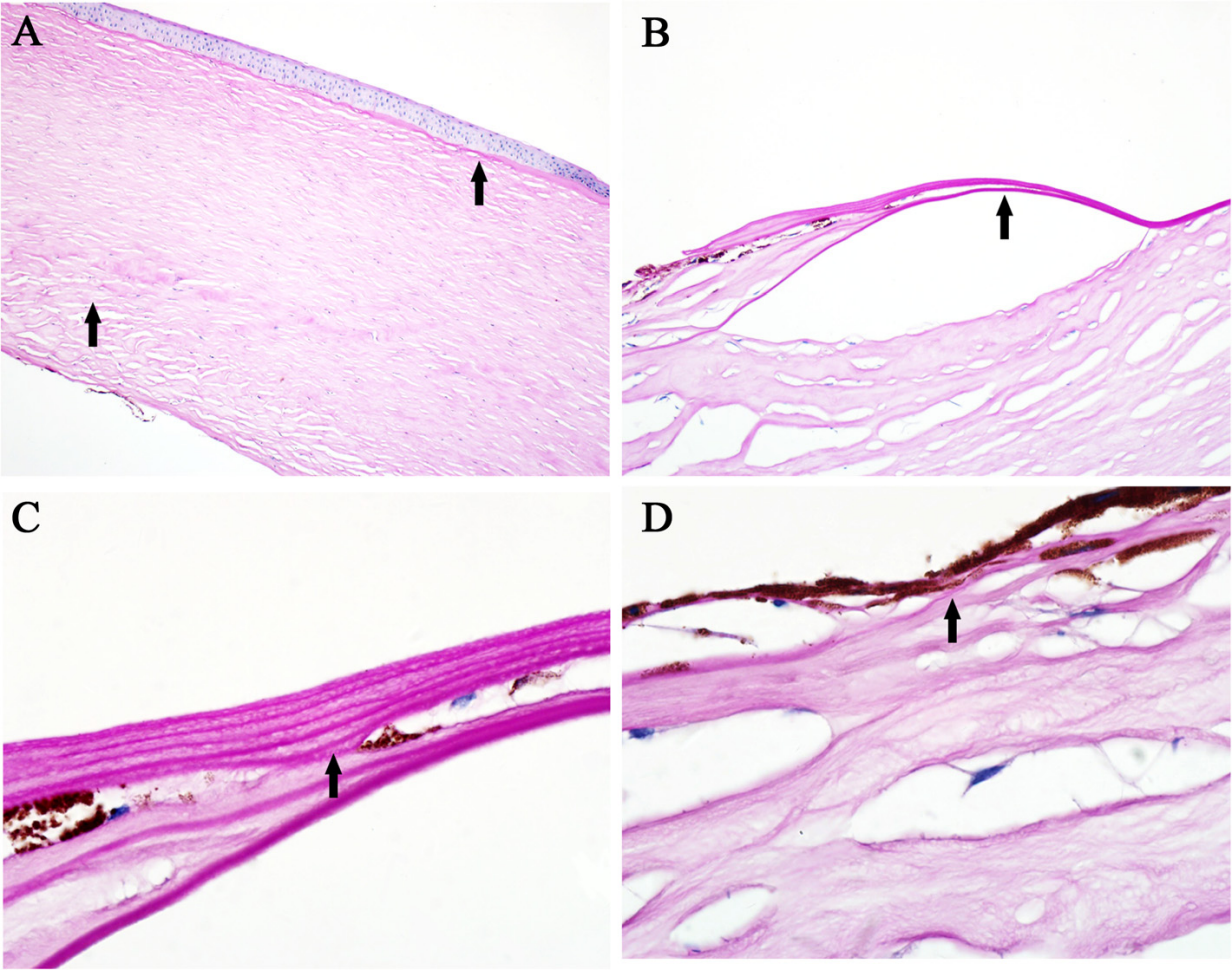
Histological photographs of the cornea. **a** Bowman’s membrane was completely absent in the middle area of the cornea. The central internal stroma was hypoplastic and the fibers were extremely disarranged without vessels. The endothelium membrane was entirely absent from the cornea. (Periodic Acid-Schiff stain, original magnification × 10). **b** An abnormal thickness of Descemet’s membrane was present with the “multiple-layer” structures at the peripheral part of the defect. (Periodic Acid-Schiff stain, original magnification × 40). **c** The “multiple-layer” structures were found in the Descemet’s membrane and pigmented tissues could be found inside the “layers”. (Periodic Acid-Schiff stain, original magnification × 100). **d** The pigmented cells situated within the cornea stroma and inserted anteriorly with adhesion to the posterior part of the cornea. Absence in Descemet's membrane in the peripheral synechiae area were shown. (Periodic Acid-Schiff stain, original magnification × 100)

Histopathologic Procedures were performed on the host corneal button, including Hematoxylin-Eosin stain and Periodic Acid-Schiff stain, to obtain the pathologic features of the cornea sections. All the sections were examined by light microscopy. On gross examination, the cornea (7 mm × 8 mm) was densely opacified centrally. Histological section showed considerable cornea thinning centrally (Fig. 3a), besides which the defects in the endothelium, Descemet's membrane, cornea stroma and Bowman’s membrane were also observed at the corneal defect. Bowman’s membrane was completely absent in the middle area of the corneal button and no vessels were found in the anterior stroma (Fig. 3a). The central internal stroma was hypoplastic and the fibers were extremely disarranged. (Fig. 3a). Most importantly, an abnormal thickness of Descemet’s membrane was present with the “multiple-layer” structure in the peripheral part of the cornea (Fig. 3b, c). Pigmented tissues could be discovered inside these “layers” of Descemet’s membrane (Fig. 3c). Furthermore, the pigmented cells situated within the cornea stroma and inserted anteriorly with adhesion to the posterior part of the cornea (Fig. 3d). Histopathologic examination also detected a large absence of the Descemet's membrane in the synechiae area (Fig. 3d) and the endothelium membrane was completely absent from the cornea (Fig. 3a).

Taken together, the major histopathological findings in the patient include “multiple-layer” structure of the Descemet's membrane in the peripheral area, the absence of cornea endothelium, hypoplasia of the cornea stroma, absence of Bowman’s membrane, and the attachment of iris to the cornea.

## Discussion

The incidence of ocular malformation in newborns ranges from 3.3 to 6.0 per 10,000 births [3] and Peters anomaly acounts for the largest proportion of the anterior segment dysgenesis. An estimate of 44-60 cases of Peters anomaly is reported in the United States annually [4]. One of the essential features of Peters anomaly is congenital central corneal opacity with defects in the posterior stroma, Descemet’s membrane, and endothelium. These conditions are characterised by both autosomal dominant and recessive patterns of inheritance with variable expressivity [5].

Clinical features of Peters anomaly are diverse, from mild to severe. The most common ocular abnormalities associated with Peters anomaly were glaucoma (20 %), microphthalmia (18 %), and colobomas (6 %) [6]. A study by Traboulsi at al [7] discovered that 25 % of their patients had microphthalmia and/or chorioretinal colobomas and 48 % had associated congenital systemic malformations. More recently, Peters anomaly was subdivided into 2 types [1]. Type I is characterized by the central corneal opacity with iridocorneal adhesions, in which the lens may or may not be cataractous, whereas type II is characterized by the central corneal opacity with cataracts or corneorlenticular adhesions. Peters Plus Syndrome (PPS) [8] comprises anterior chamber defects along with other systemic anomalies. To prevent amblyopia, the earlier treatment is performed, the better the results are. There are a variety of surgical options available for treating Peters anomaly. Peripheral optical iridectomy may be carried out for patients with a clear peripheral cornea. As for patients with bilateral visually disabling corneal opacity, penetrating keratoplasty is recommended. [9] It has been suggested that early keratoplasty for congenital corneal opacities might improve the visual outcome [9]. However, some studies showed a wide range of graft failure rates at 1 year ranging from 22 to 67 % [9–11]. Due to the high prevalence of PKP graft failure, some doctors chose to use the Boston Type I Keratoprosthesis (KPro-1) in these patients [12].

In Peters anomaly, the corneal opacity is either central or paracentral and it usually does not exhibit vascularization [13]. Histopathology is often diagnostic. Previous histopathologic findings of Peters anomaly described the defects and absence in the endothelium, Descemet’s membrane, posterior stroma and the Bowman’s membrane of the cornea, which are consistent with our pathologic findings of this patient. However, the “multiple-layer” structure of the Descemet’s membrane has not been reported as in association with abnormalities of the cornea tissues in Peters anomaly. The cause of this unusual structure, which may be due to genetic factors, environmental factors, or both, has not been clearly identified in pathologic studies so far.

Peters anomaly is a genetic disease affecting multiple anterior segments, which is known to be sporadic, but sometimes inherited [1]. Several genetic mutations [1, 2, 9] act in concert to specify a population of mesenchymal progenitor cells, as they migrate anteriorly around the embryonic optic cup [1, 2, 9]. Waring [14] demonstrated various developmental mechanisms for Peters anomaly, including faulty separation of the lens vesicle from the surface ectoderm, primary abnormal migration of neural crest cells into the cornea, and intrauterine corneal inflammation. Development of the ocular anterior segment depends largely on periocular mesenchyme cells, which are derived predominantly from neural crest cells (NCC) [15]. Specific and differential cell adhesion is expected to be instrumental in induction, migration, and differentiation of NCC. Tian et al [15] demonstrated that NCC-specific inactivation of p120 catenin mice perturbs the proper development of certain eye structures and causes anterior segment dysgenesis, including adhesion of the iris to the cornea, hypoplasia of iris and ciliary body, and dysgenesis of cornea, trabecular meshwork, and Schlemm’s canal.

Peters anomaly shows diverse clinical/pathologic features and visual outcomes. Consistent with previous findings, our case has discovered diverse histopathologic features of the cornea layers, especially in the Descemet's membrane. However, little is known of the development of the abnormal Descemet's membrane in patients with Peters anomaly. Although this observation does not imply causation and there still are many “unknown areas” in the pathological diagnosis of this special structure, such pathological findings need to be reported to enhance further characterization and it may be of interest to others who have observed similar associations. Further study of the disease may be required.

## Conclusion

The “multiple-layer” structure of the Descemet’s membrane described in our case has not been reported as associated with abnormalities of the cornea tissues in Peters anomaly. Such pathological findings need to be reported to enhance further understanding of the special structure of Descemet's membrane as an abnormality during embryogenesis and neural crest cell differentiations.

### Consent

Written informed consent was obtained from the patient and her father for publication of this case report and any accompanying images. A copy of the written consent is available for review by the editor of this journal.
